# Editorial: Exploring heavy work investment: multidimensional constructs and work outcome variance

**DOI:** 10.3389/fpsyg.2025.1656269

**Published:** 2025-07-28

**Authors:** Christian Vandenberghe, Aharon Tziner, Julio César Acosta-Prado

**Affiliations:** ^1^HEC Montreal, Management Department, Montreal, QC, Canada; ^2^Tel-Hai College, Upper Galilee, Israel; ^3^Universidad Externado de Colombia, Bogotá, Colombia

**Keywords:** heavy work investment, workaholism, engagement (involvement), commitment, health outcomes

Heavy Work Investment (HWI) is a broad, multidimensional construct that refers to the sustained and significant investment of time and energy in the work domain. In their pioneering work, Snir and Harpaz (2012) emphasized that HWI is not inherently positive or negative; rather, its consequences depend on the type of investment, underlying motivations, and contextual factors. Research has primarily identified two forms of HWI: workaholism, characterized by an uncontrollable drive to work excessively and compulsively (Clark et al., 2016; Schaufeli et al., 2008), and work engagement, defined as a positive, fulfilling psychological state marked by vigor, dedication, and absorption (Bakker et al., 2014).

Given its multidimensional nature and context-dependent outcomes, HWI can be approached from various angles, including its sustainability over time, the personality traits involved, and its broader organizational context. It can be linked to a wide range of outcomes and related constructs, such as engagement and commitment. As such, HWI continues to inspire diverse lines of research. Notable areas of inquiry include the attitudinal and behavioral consequences of HWI over time, its health-related implications, impact on work-family conflict, gender differences in sensitivity to HWI, and its association with career success. Furthermore, the prevalence and role of workaholism within the general population remain topics of ongoing interest. This Research Topic brings together a set of articles that explore these various facets of HWI, offering a comprehensive perspective on how it can be studied, understood, and addressed, highlighting its significance for today's working population.

Andersen et al. conducted a review and meta-analysis estimating the prevalence of workaholism, summarizing data from 53 studies (71,625 participants) across 23 countries. With an overall pooled prevalence of 15.2%, the analysis reveals that roughly one in seven employees may be affected by workaholism. Meta-regression showed that studies using nationally representative samples reported significantly lower prevalence (around 9.8%), while non-representative samples yielded higher estimates. Studies using the Dutch Work Addiction Scale (DUWAS) reported higher rates compared to the Bergen Work Addiction Scale (BWAS). Significant heterogeneity persisted in the results, with only 29% of variance explained, indicating the need for future research to account for additional factors like culture, temporal trends, and workforce demographics. The article concludes by emphasizing the public health relevance of workaholism, urging better-designed representative studies to inform prevention and intervention strategies.

Escudero-Guirado et al. present a literature review of 83 studies examining HWI with a specific focus on gender dynamics and workplace flexibility. Employing bibliometric and co-word analyses, the authors grouped research into six thematic clusters, identifying work-family conflict as the central theme. The review indicates that incorporating a gender lens, particularly for women in societies like China and Japan, shifts emphasis from job demands to job resources, especially flexibility, which supports work-life balance. The study highlights a growing focus on organizational support mechanisms that facilitate resource-based rather than demand-based approaches to HWI among women. The paper identifies cultural contexts and workplace flexibility as underexplored topics, and advocates for future cross-cultural and gender-sensitive research in organizational support and flexible work arrangements.

Aziz and Covington examine workaholism as distinct from work addiction and work engagement, tracing its conceptual roots, measurement, and differentiation from other constructs. The authors summarize two decades of research linking workaholism to negative health outcomes, including poor sleep, heightened stress, musculoskeletal complaints, and burnout. The review highlights evidence associating workaholism with cardiovascular risk (e.g., hypertension, dyslipidemia) and metabolic challenges, noting that excessive overtime and work stress correlate with coronary heart disease and impaired lipid profiles. As many studies rely on self-report, cross-sectional data, the authors recommend future longitudinal research to establish causality, exploring biological pathways such as inflammation, glucose regulation, physical activity, and recovery behaviors. The paper also argues for an integration of organizational psychology and biomedical research to better understand workaholism's role in chronic diseases.

Afota et al. conducted a three-wave longitudinal study among business alumni to explore how three forms of HWI (workaholism, work engagement, and affective organizational commitment) relate to self-concept levels and predict work outcomes over time. They found that workaholism was uniquely associated with both the individual and collective self-concepts, while engagement and commitment were linked solely to the collective self-concept. Regarding outcomes, workaholism, but not the other two constructs, predicted later increases in work hours, perceptions of role overload, and the likelihood of depression. In contrast, work engagement served a protective role, reducing the risk of depression and emotional exhaustion over time, while affective commitment had no significant influence on these outcomes.

Turan-Torun et al. explore how digital leadership influences employee job performance, job satisfaction, and career satisfaction, with HWI (time commitment and work intensity) as mediators. The study surveyed 393 employees from Turkish IT SMEs. Results show that digital leadership positively affected all three outcomes. Work intensity partially mediated these effects while time commitment only mediated the effect on job performance. This suggests that time investments can lead to stress and burnout, while intensity-driven efforts benefit employees under digital leadership. The paper acknowledges limitations, such as convenience sampling in a specific sector and country, and calls for broader research to deepen understanding of digital leadership's mechanisms.

Oldewage and Jonck investigated how Dark Triad personality traits (narcissism, Machiavellianism, and psychopathy) interacted with career interests to predict subjective and objective career success among 300 South African professionals. They found that the association between narcissism and higher career success is amplified for individuals with enterprising interests, while Machiavellianism was linked to better success when social interests were high. In contrast, psychopathy showed limited predictive value for career outcomes regardless of interest type. The study situates its findings within person-environment fit theory, highlighting that Dark Triad traits can be leveraged adaptively depending on one's vocational interests. The authors acknowledge limitations to their study, including cross-sectional design and self-report measures, and call for longitudinal and multi-method approaches to clarify causal pathways.
